# Mesenchymal Stem Cells from Human Umbilical Cord Express Preferentially Secreted Factors Related to Neuroprotection, Neurogenesis, and Angiogenesis

**DOI:** 10.1371/journal.pone.0072604

**Published:** 2013-08-22

**Authors:** Jui-Yu Hsieh, Hsei-Wei Wang, Shing-Jyh Chang, Ko-Hsun Liao, I-Hui Lee, Wei-Shiang Lin, Chun-Hsien Wu, Wen-Yu Lin, Shu-Meng Cheng

**Affiliations:** 1 Institute of Microbiology and Immunology, National Yang-Ming University, Taipei, Taiwan; 2 Department of Obstetrics and Gynecology, Hsin-Chu Mackay Memorial Hospital, Hsin Chu, Taiwan; 3 Cancer Research Center & Genome Research Center, National Yang-Ming University, Taipei, Taiwan; 4 Departments of Education and Research, Taipei City Hospital, Taipei, Taiwan; 5 Department of Neurology, Neurological Institute, Taipei Veterans General Hospital, Taipei, Taiwan; 6 Institute of Brain Science, National Yang-Ming University, Taipei, Taiwan; 7 Division of Cardiology, Department of Internal Medicine, Tri-Service General Hospital, National Defense Medical Center, Taipei, Taiwan; Georgia Regents University, United States of America

## Abstract

Mesenchymal stem cells (MSCs) are promising tools for the treatment of diseases such as infarcted myocardia and strokes because of their ability to promote endogenous angiogenesis and neurogenesis *via* a variety of secreted factors. MSCs found in the Wharton’s jelly of the human umbilical cord are easily obtained and are capable of transplantation without rejection. We isolated MSCs from Wharton’s jelly and bone marrow (WJ-MSCs and BM-MSCs, respectively) and compared their secretomes. It was found that WJ-MSCs expressed more genes, especially secreted factors, involved in angiogenesis and neurogenesis. Functional validation showed that WJ-MSCs induced better neural differentiation and neural cell migration *via* a paracrine mechanism. Moreover, WJ-MSCs afforded better neuroprotection efficacy because they preferentially enhanced neuronal growth and reduced cell apoptotic death of primary cortical cells in an oxygen-glucose deprivation (OGD) culture model that mimics the acute ischemic stroke situation in humans. In terms of angiogenesis, WJ-MSCs induced better microvasculature formation and cell migration on co-cultured endothelial cells. Our results suggest that WJ-MSC, because of a unique secretome, is a better MSC source to promote *in vivo* neurorestoration and endothelium repair. This study provides a basis for the development of cell-based therapy and carrying out of follow-up mechanistic studies related to MSC biology.

## Introduction

Stem cells have attracted much attention due to their unique biological behaviour and potential clinical usage. Mesenchymal stem cells (MSCs) are able to improve outcomes when there are bone and other tissue defects, including osteogenesis imperfecta [1], infarcted myocardium [2,3], and brain injury [4]. In rats, administration of MSCs 1 day or 7 days after stroke reduces neurological functional deficits [5]. Clinical phase I trials in patients with chronic ischemic stroke [6] or with spinal cord injury [7] have suggested that intravenous autologous MSC transplantation reduces long-term disability in the treated patients and caused no serious adverse events related to MSC transplantation during follow-up. Recent studies have suggested that MSC-based therapy of brain injury enhances not only neurogenesis but also angiogenesis [8].

MSCs can be harvested from many tissues, including bone marrow, cord blood, umbilical cord and adipose tissue [9–11]. MSCs from Wharton’s jelly of the umbilical cord (WJ-MSCs) are multipotent and are able to give rise to various types of cells, including osteocytes, adipocytes and chondrocytes [12,13]. Although the immunophenotypic profiles of MSCs from different tissues are similar [9,13,14], the disparate characteristics, including proteomics, genomics, and functionality, of MSCs from different sources have been described and compared in detail for better applying MSCs clinically [15–18]. A quantitative proteomic and transcriptomic comparison of human mesenchymal stem cells from bone marrow and umbilical cord vein showed that MSCs from both tissues shared high similarity in metabolic and functional processes relevant to their therapeutic potential, especially in the immune system process, response to stimuli, and processes related to the delivery of the MSCs to a given tissue, such as migration and adhesion. Hence, our results support the idea that the more accessible umbilical cord could be a potentially less invasive source of MSCs [19].

Transplanted MSCs not only directly differentiate into neurons and endothelial cells after injection [20,21], but also secrete a broad repertoire of trophic and immunomodulatory cytokines, generally referred to as the MSC secretome, which has considerable potential for the treatment of various diseases such as cardiovascular disease and brain damage *via* an induction of endogenous neuro-protection, neurogenesis and angiogenesis [8,22,23]. As a result, the MSC secretome has considerable potential for the treatment of central nervous system (CNS) degeneration and ischemic heart diseases [23,24]. However, harnessing this MSC secretome for meaningful therapeutic outcomes is challenging due to the limited knowledge and control of cytokine production following their transplantation. For example, the secretome of bone marrow mesenchymal stem cells-conditioned media varies with time and drives a distinct effect on primary neurons and glial cells [25]. Addressing the compositions and variations in secretome of MSCs from different sources or expanded *ex vivo* under different conditions (e.g., hypoxia [26] or serum deprivation [27]) will eventually benefit the future application of MSCs in regenerative medicine.

Secretomes of stem cells from different anatomic resources also vary: for example, comparative analysis of paracrine factor expression in human MSCs derived from bone marrow, adipose (ASCs), and dermal tissue [dermal sheath cells (DSCs) and dermal papilla cells (DPCs)] showed that vascular endothelial growth factor-A (VEGF-A), angiogenin, basic fibroblast growth factor (bFGF/FGF2), and nerve growth factor (NGF) were expressed at comparable levels among the MSC populations examined, while ASCs expressed significantly higher levels of insulin-like growth factor-1, VEGF-D, and interleukin-8. Functional assays examining *in vitro* angiogenic paracrine activity showed that ASCs induced better tubulogenesis compared with DPCs, with VEGF-A and VEGF-D being 2 major factors [28]. The variation in paracrine factors of different MSC populations thus contributes to different levels of repair activity. MSCs from human umbilical cord Wharton jelly or adipose tissue act differently on central nervous system derived cell populations, in which WJ-MSCs have the better impact on the metabolic viability and cell density of primary hippocampal neurons [29]. Nevertheless, factors that promote the stronger effect of the WJ-MSC conditioned media in neuronal survival are still to be identified.

Here, we aim to investigate the secretome patterns, as well as paracrine neuro-protection, neurogenesis and angiogenesis effects, of BM- and WJ-MSCs. Our results provide a better understanding of the characteristics of these two types of MSCs and this will allow a better and more logical application of these MSC types in a clinical situation. 

## Materials and Methods

### Cell culture

#### Human mesenchymal stem cells

MSCs from healthy individuals were isolated from the Wharton’s jelly part of the umbilical cord; in all cases the donors provided written informed consent. The current study complies with the Helsinki Declaration. The tissue sample analysis was approved by the Institutional Review Board of Mackay Memorial Hospital, Taiwan. MSCs from Wharton’s jelly were collected as published previously [30]. Bone marrow MSCs were purchased from PromoCell (Heidelberg, Germany). All MSCs were cultured for less than eight passages in MesenCult® medium (StemCell Technologies, USA) in the presence of 5% CO_2_.

#### Cell lines

Mouse Neuron-2a (N2a) neuroblastoma cells were cultured in DMEM supplemented with 10% FBS. Human HMEC1 microvascular endothelial cells were cultured in endothelial cell growth medium MV (PromoCell, Germany).

### Array probe preparation and data analysis

Total RNA collection, cRNA probe preparation, array hybridization and data analysis were carried out as previously described [31,32]. A heatmap was created by the dChip software program (http://biosun1.harvard.edu/complab/dchip/). Gene annotation was performed by the ArrayFusion web tool (http://microarray.ym.edu.tw/tools/arrayfusion/) [33], and gene enrichment analysis was performed by DAVID Bioinformatics Resources 2012 (http://david.abcc.ncifcrf.gov/) [34]. All microarray data in this study have been deposited to the NCBI GEO database with an Accession number: GSE48022.

### RNA isolation and real-time PCR

Total mRNA were extracted by the RNeasy mini kit (Qiagen, Germany), and 100 ng to 1 µg of total RNA were subjected into reverse transcription using a First cDNA Synthesis kit (Fermentas, USA). For quantitative real-time PCR analysis, human pre-messenger RNA sequence was obtained from the NCBI (National Center for Biotechnology Information) AceView program (www.ncbi.nlm.nih.gov/AceView/). All primers were designed to cross introns using the Primer3 website (http://frodo.wi.mit.edu/cgi-bin/primer3/primer3_www.cgi/) or Primer Express software (Applied Biosystems, CA, USA). Thermodynamics and primer specificity analysis were performed by the Vector NTI suite (Invitrogen, CA, USA) and the NCBI reverse e-PCR program (http://www.ncbi.nlm.nih.gov/sutils/e-pcr/reverse.cgi/). Real-time PCR reactions were performed using Maxima^TM^ SYBR Green qPCR Master Mix (Fermentas, USA), and the specific products of the PCRs were detected and analyzed using a StepOne^TM^ sequence detector (Applied Biosystems, USA). The expression level of each gene was normalized against the expression level of glyceraldehyde 3-phosphate dehydrogenase (GAPDH). All the primer sequences are listed in Table S1.

### Cell migration assays

All cell migration assays were performed in 24-well Transwell plates (Corning Inc., NY, USA). A total of 3 × 10^4^ MSCs in 600 μl of MesenCult® medium was added to the lower compartment. After 1 day, 7.5 × 10^4^ N2a cells or 5 × 10^4^ HMEC1 in 100 µl of original culture medium were seeded into the upper Transwell inserts. After 6-12 hours, the cells that had migrated across the membrane were stained with Hoechst 33342, and analyzed by fluorescent microscopy.

### Preparation of conditioned medium and enzyme-linked immunosorbent assay (ELISA)

A total of 10^5^ MSCs cultured in MesenCult® medium were plated in 6-well plates. After 1 day, the medium was changed with fresh MesenCult® medium and the MSC conditioned medium was collected 1 day later.

The levels of PGF and CXCL5 in BM and WJ conditioned medium were quantified ELISA (RayBiotech, Norcross, USA) according to the manufacturer’s instructions.

### N2a differentiation and HMEC1 tube formation

For N2a differentiation, 4 × 10^4^ N2a cells were plated in 6-well plates. After 1 day, N2a cells were treated with MSC-conditioned medium for 4 days. Differentiated N2a cells were collected for immunoblotting and immunofluorescent analysis. For HMEC1 tubule formation, 50 µl of thawing Matrigel (Cultrex Basement membrane extract, Trevigen Inc, Gaithersburg, MD) was placed in 96-well plate for 1 hour at 37℃. HMEC1 cells were harvested and placed on the Matrigel in 100 µl MSC-conditioned medium for 4 hours. Tubule formation was monitored by inverted light microscopy (100x).

### Oxygen-glucose deprivation (OGD)

#### Primary cortical cultures

All animal procedures were approved by Institutional Animal Care and Use Committee (IACUC) of National Yang-Ming University, Taiwan (NYMU), and animals were kept in accordance with the guidelines of Laboratory Animal Center (LAC) at NYMU. Neuronal-enriched cortical cultures were obtained from fetal Sprague-Dawley rats based using a protocol described previously [35]. The pregnant rats are decapitated after isoflurane (10ml/kg body weight) anesthesia. Cortices were dissected mechanically from embryonic rat brains at E17; these were then triturated, filtered, centrifuged in HBSS, and cultured at 37°C in Neurobasal medium (Invitrogen) supplemented with 10% B27 (Invitrogen), 100 U/ml penicillin and 100 mg/ml streptomycin with 5% CO_2_. For the OGD, the cultures were placed into a hypoxic incubator (Thermo Scientific model 3130) on day 4 at 1% O_2_ with a gas mixture of 95% N2/5% CO_2_, and the medium was replaced with glucose-free DMEM (Gibco 11966-025) for 24 hours. Afterwards, the cultures were returned to a normoxic incubator, and the medium was replaced with Neurobasal medium with B27.

#### MSC and cortical cells co-culture

For trans-membrane co-cultures, BM-MSCs or WJ-MSCs were plated on membrane inserts at a density of 5 x 10^4^ cells per 12 mm Millicell Cell Culture insert (Millipore, with 0.4 µm pores) and placed above the cortical cultures after OGD 3 days before fixation (n = 4 per condition in each group).

Cortical cell death and apoptosis was evaluated using Propidium iodide (PI) staining and *in Situ* Cell Death Detection Kit (TUNEL), according to manufacturer’s instructions (Roche). For both assays, cells were co-stained with DAPI. The results were expressed as a percentage of the number of PI-positive or TUNEL-positive cells to the total number of cells stained with DAPI (n = 4).

### Immunofluorescent and immunoblotting assays

For N2a immunofluorescence, cells were fixed 20 minutes by fixation solution (4% paraformaldehyde in PBS) at room temperature, and then blocked with blocking solution for 30 min. Blocking solution consisted of 0.05% Triton X-100, 5% bovine serum albumin in PBS. Cells were incubated with primary antibodies for 1 hour at room temperature, and secondary antibodies for another 1 hour. Antibodies used were monoclonal mouse anti-β-tubulin III (1:200, MAB1637; Chemicon, USA) and FITC-conjugated goat anti-mouse IgG. For rat cortex cells immunofluorescence, cells were placed on poly-D-lysine hydrobromide (PDL, P6407, Sigma)-coated coverslips, fixed in 4% paraformaldehyde for 20 min at room temperature, washed, and incubated in a blocking solution containing 0.15% Triton X-100 and 5% bovine serum albumin (BSA) in 0.1 M PBS at room temperature for 1 hour. The samples were then incubated with primary antibodies against MAP2 (1:75, Mab3418, Chemicon, USA) and GFAP (1:500, Z0334, Dako) at 4^°^C overnight, washed and incubated with secondary antibodies conjugated with Alexa 488 and/or Cy3 (Jackson ImmunoResearch, 1:200) at room temperature for 1 hour in the dark. The coverslips were mounted with VECTASHIELD mounting medium containing DAPI (H-1200, Vector Laboratories) and photographed using a confocal laser-scanning microscope (Olympus FV1000 and ZEISS LSM 700). The quantification of the cell counts and the total length of neurite outgrowth were obtained using Image Pro Plus software V 6.3 (Media Cybernetics, Inc., USA). Western blotting was performed as previously published [36] using the following antibodies: mouse anti-β-actin (A5441, Sigma, USA), rabbit anti-NEFL (AB1983, Chemicon, USA), and mouse anti-TUBB3 (MAB1637, Chemicon, USA).

## Results

### Functional group analyses revealed distinct secreted factor patterns between BM-MSCs and WJ-MSCs

**Figure 1:**
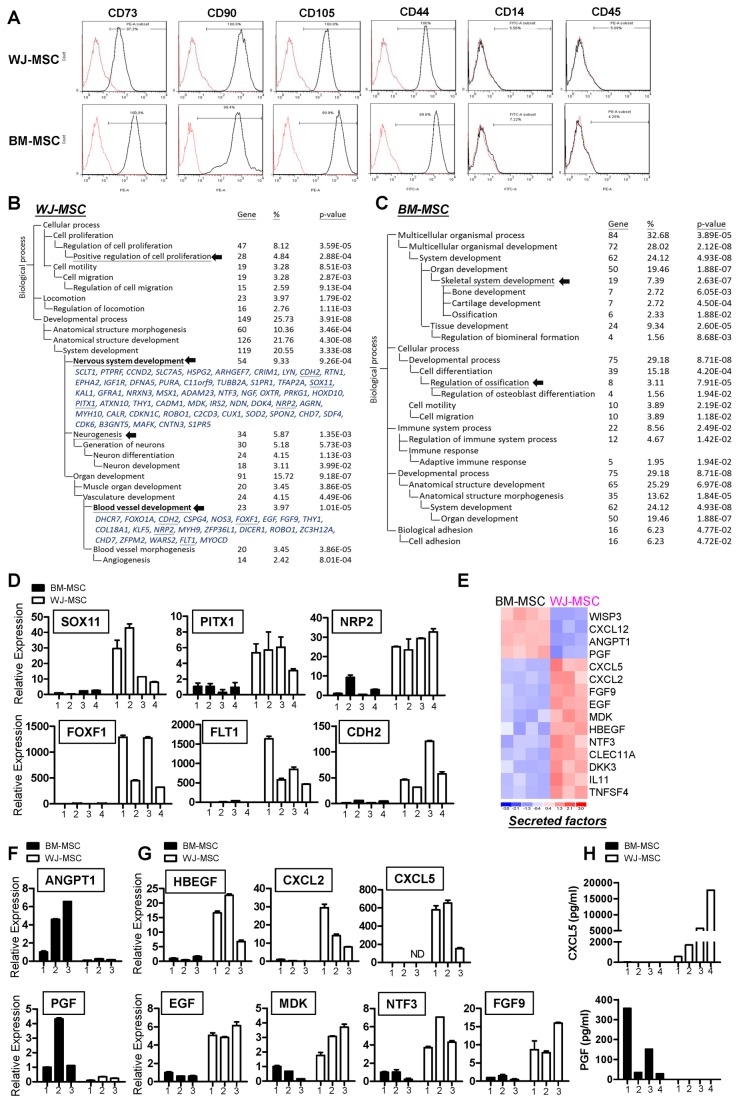
Differential gene expression between WJ-MSCs and BM-MSCs. (**A**) Flow cytometry analysis of WJ-MSCs and BM-MSCs. (**B**–**C**) A total of 1096 probe sets (q ≦ 10^-3^) differentiating WJ-MSCs and BM-MSCs were filtered out and 762 WJ-MSC probe sets (B) and 334 BM-MSC probe sets (C) were subjected to DAVID (http://david.abcc.ncifcrf.gov/) analysis. These categories were selected using the Biological Process organizing principle via the Gene Ontology project (http://www.geneontology.org/). The number of genes, as well as *p* values, for categories that are significantly (*p*<0.05) over-represented are listed. The terms indicated by arrows are discussed in the text. Genes involved in nervous system development and blood vessel development are listed, and RT-qPCR verified genes are underlined. (**D**) RT-qPCR validation of the relative expression levels of the neurogenic-related and angiogenic-related genes in the two MSC subtypes. Mean expression levels of the target genes were compared to that of the GAPDH control. Each bar represents a different individual. Results were expressed as the mean ± standard deviation (SD). (**E**) A heatmap showing BM-MSC and WJ-MSC-specific secreted factors. (**F**–**G**) Validation of array data by RT-qPCR. *ANGPT1* and *PGF*, which are up-regulated in BM-MSCs (**F**), as well as various WJ-MSC abundant genes (**G**), were examined. (**H**) The secretion levels of CXCL5 and PGF in the conditioned medium of BM-MSC and WJ-MSC were quantified using enzyme-linked immunosorbent assays. Each bar represents the protein concentration of independent donors.

To access the functional differences between human MSCs from different anatomical sources, primary BM-MSCs and WJ-MSCs were collected. Both MSCs were positive for CD73, CD90, CD105 and CD44, while lacking of hematopoietic markers CD14 and CD45 (Figure 1A). Gene expression profiles of both types of MSC were carried out in at least triplicate using whole-genome chips. Genes that were differentially expressed when the two types of cell were compared (the molecular signature) were identified by statistical pipeline [31,32]. A total of 762 probe sets were abundantly over-expressed in WJ-MSCs compared to BM-MSCs, while another 334 probe sets were abundantly over-expressed in BM-MSCs compared with WJ-MSCs (with a positive false discovery rate (pFDR) threshold *q*<0.001). The filtered gene lists are presented in Table S2.

The gene signatures provided a limited indication of the functional differences associated with these two MSC subtypes. To gain more insight into the functional consequences of these differential gene expression patterns and to provide quantitative evidence, the signature genes were subjected to a Gene Ontology (GO) database search [37] to find statistically over-represented functional groups within gene lists. The DAVID web-based tool was applied for this task [34]. The GO categories of the biological processes that were statistically overrepresented (*p*<0.05) are shown in Figure 1B-C. The most predominant of these overrepresented processes in WJ-MSCs include those pertaining to cell proliferation, neurogenesis, nervous system development and blood vessel development (Figure 1B; indicated by arrows). In contrast, the principal overrepresented processes associated with BM-MSCs include those related to ossification and skeletal system development (Figure 1C). In WJ-MSCs, neurogenesis transcriptional factors such as *SOX11* [38] and *PITX1* [39], as well as transcriptional factors associated with angiogenesis such as *FOXF1* [40,41], were highly expressed, and these findings were confirmed by RT-qPCR (Figure 1D). Receptors involved in neurogenesis and angiogenesis, including *CDH2, NRP2* and *FLT1*, were also more abundant in WJ-MSCs (Figure 1D).

As mentioned in the *Introduction*, the main task of this study is to explore the secretome of different MSCs, as well as to compare the functional differences of conditioned media of MSCs. We therefore focused on the differential expression of secreted factors between BM-MSCs and WJ-MSCs. Each of the two MSCs secretes unique cytokines, chemokines and growth factors: known neurogenic cytokines, such as NTF3, EGF and MDK, were found to be highly expressed in WJ-MSCs, and both MSC types expressed a variety of, but different, angiogenesis-related growth factors (Figure 1E). Of note, these data only showed the differential expression of these secreted factors between different MSCs. Both MSCs may still express these factors, just at different amounts. RT-qPCR analyses verified the differential expression of the up-regulated BM-MSC genes, namely *PGF* and *ANGPT1* (Figure 1F), and the WJ-MSC up-regulated genes, namely *HBEGF*, *CXCL5*, *CXCL2*, *EGF, FGF9, MDK* and *NTF3* (Figure 1G). Levels of secreted proteins were also monitored in culture supernatants (conditioned medium) collected from BM-MSCs and WJ-MSCs using enzyme-linked immunosorbent assay (ELISA). WJ-MSCs produced significantly higher amount of CXCL5 in their conditioned medium (570.3-17622.4 pg/ml) as compared with BM-MSCs, and the secreted level of PGF was higher in BM-MSC conditioned medium (28.6-356.7 pg/ml) (Figure 1H).

### BM-MSCs and WJ-MSCs have different neural induction abilities

**Figure 2:**
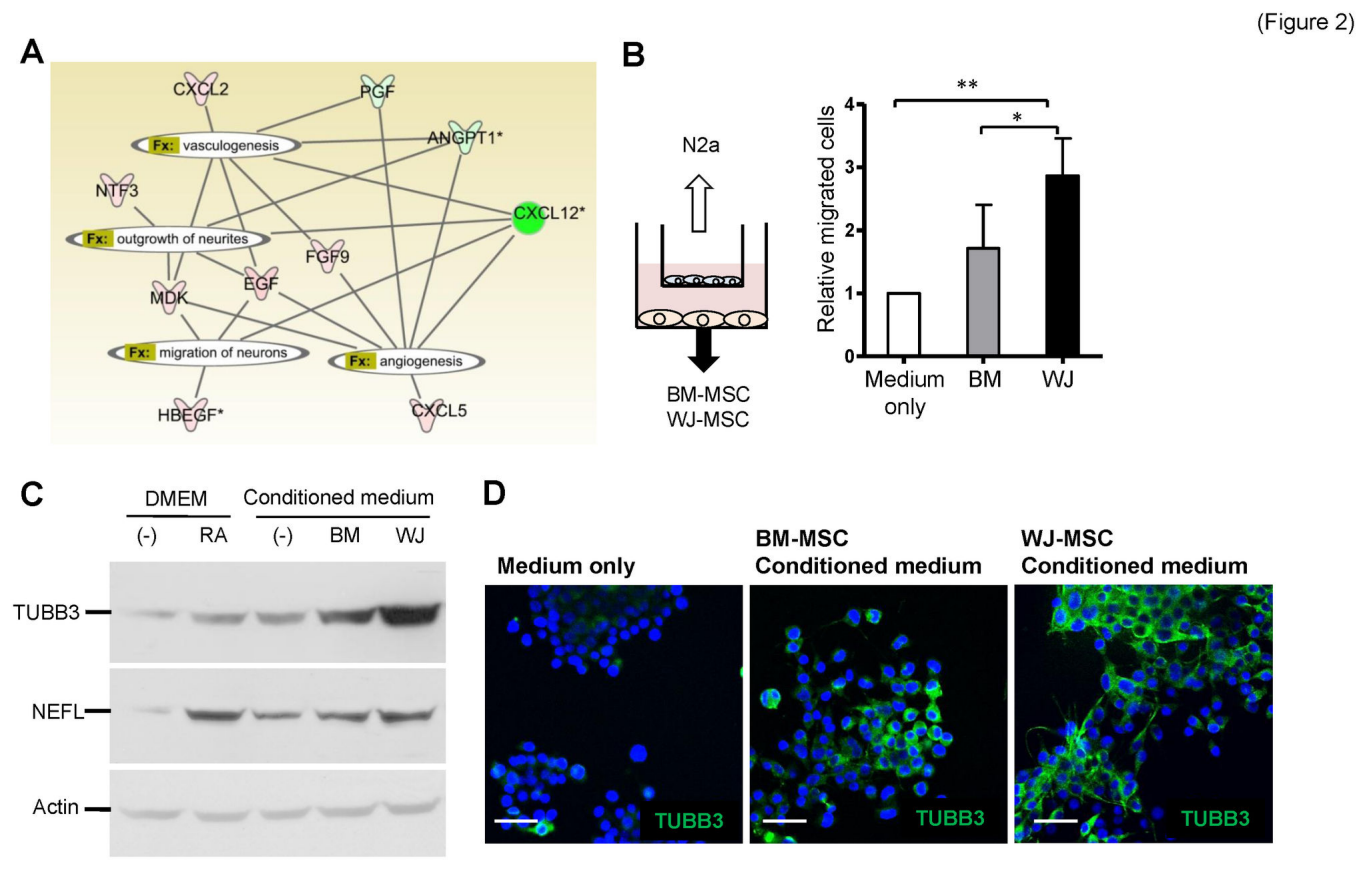
Higher neural induction ability of WJ-MSCs. (**A**) An interaction network of secreted factors. Factors that pertained to angiogenesis, vasculogenesis, neurite outgrowth, and neuron migration are connected. Genes in red are abundant in WJ-MSCs and genes in green are abundant in BM-MSCs. (**B**) Differential chemotaxis effects of WJ-MSCs and BM-MSCs. MSCs cultured in MesenCult® medium were seeded in the lower part of Tranwell plates, while N2a cells were placed in the upper chambers (illustrated in left panel). Migrated N2a cells to the other side of the membrane were stained with Hoechst 33342 and counted. Data are mean ± SD (right panel; *p<0.05, **p<0.01). (**C**–**D**) Induction of N2a neural differentiation by MSCs. N2a cells were cultured with MSC conditioned medium, medium only (negative control) or retinoic acid (RA; positive control) for 4 days before the cellular lysates were subjected to Western blotting analysis (C) or the cells were fixed for immunofluorescence staining (D). Neural markers TUBB3 and NEFL were analyzed. Cell nuclei were stained with DAPI. Scale bars: 50 μm.

To gain more insights into the biological impact of these differential secretomes, we utilized Ingenuity Pathway Analysis (IPA) software to evaluate growth factor functionality, specifically neurogenesis and angiogenesis. Most of the secreted factors were found to be related to neurogenesis, angiogenesis, and vasculogenesis (Figure 2A), which suggests that both MSCs possess neural and angiogenic induction abilities.

We investigated whether distinct secretomes confer different neural and angiogenic promoting abilities on the two MSCs. First we examined the chemotaxis effects of the two MSCs on neural cells. Mouse Neuro-2a (N2a) cells, which are capable of neuron differentiation and axonal outgrowth [42], were co-cultured with the two types of MSCs individually in Transwell plates for evaluating the chemotaxic effects of the MSCs on neural cells (Figure 2B, left panel). Both types of MSCs induced N2a migration, but it was clear that N2a cells moved toward the WJ-MSCs more efficiently (Figure 2B, right panel). Next we examined the ability of these two MSCs to induce N2a cell neuronal differentiation. N2a cells were cultured with the conditioned media from WJ-MSCs and BM-MSCs individually for 4 days before total proteins were collected from the cultured cells. Retinoic acid (RA) was used as a positive control for neural differentiation [43]. WJ-MSC conditioned medium induced better neural differentiation of the N2a cells, as evidenced by higher TUBB3 (β-tubulin III) and NEFL (neurofilament, light polypeptide) marker expression from the WJ-MSC conditioned medium N2a population (Figure 2C). Further substantiating this finding, immunofluorescence staining showed that there were more neurite-positive, TUBB3-positive cells after culturing N2a cells with WJ-MSC conditioned medium (Figure 2D) compared to N2a cells cultured with BM-MSC conditioned medium.

### Superior neuroprotective effect of WJ-MSC secretome on primary cortical cells injured by oxygen-glucose deprivation (OGD)

**Figure 3:**
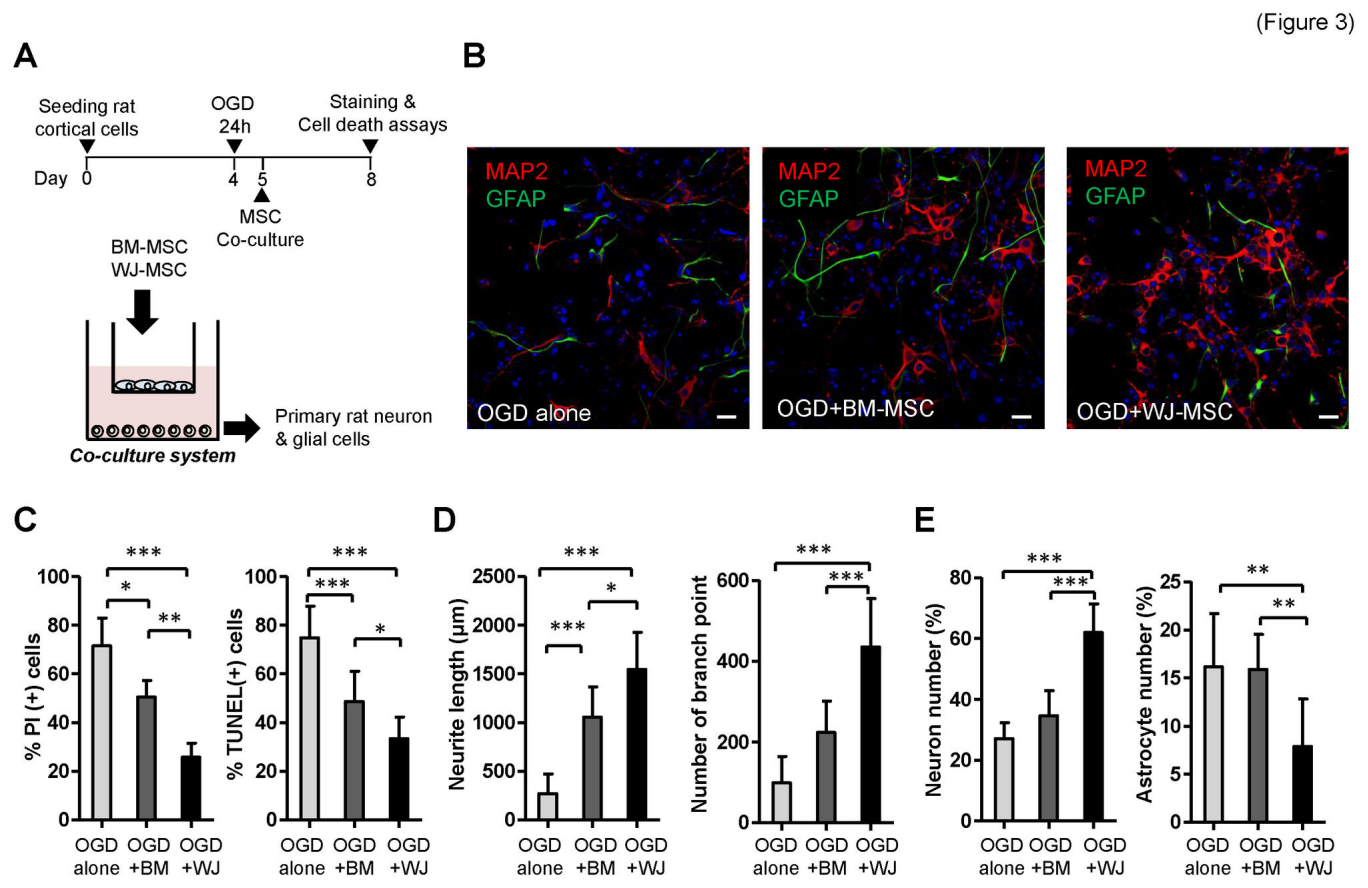
Preferential neuroprotection effects of WJ-MSCs. (**A**) Schematic representation of the transmembranous stem cell co-culture system using an oxygen-glucose deprivation (OGD) model. (**B**) Immunofluorescence staining of the rat primary cortical cells subjected to OGD alone (left), to co-culture with BM-MSCs (middle), or to co-culture with WJ-MSCs (right) at 72 hours post-OGD. Neuronal marker MAP2 is shown in red, the astroglial marker GFAP in green, and DAPI nuclear staining in blue. Scale bar: 20 µm. (**C**) Quantification of cell death and apoptosis rate using PI and TUNEL staining, respectively, at 72 hours post-OGD. *p<0.05, **p<0.01, ***p<0.001 (**D**) Quantification of total neurite length (left) and neurite branch point numbers (right). (**E**) Percentage of neuron number (left) and astrocyte number (right) after 72 hours co-cultured with BM-MSCs or WJ-MSCs post-OGD.

We next compared the neuroprotective efficacy of secreted factors from the two stem cell types using a similar trans-membranous co-culture system. OGD, a widely used *in vitro* model of ischemia, was used to mimic *in vivo* central nervous system insult (Figure 3A). The primary cortical cultures isolated from embryonic rats consisted of more than 90% of MAP2-positive neurons and approximately 2% GFAP-positive astrocytes (data not shown). After 24 hours of OGD, primary rat cortical cells were co-cultured with BM-MSCs or WJ-MSCs for 72 hours, and neuroprotection potentials of both MSCs were evaluated by immunofluorescence staining and cell death assays (Figure 3A-B). Cortical cultures were found to be severely injured after OGD, and cell death and apoptosis were increased with time to more than 70% by measuring PI positive and TUNEL positive cells (OGD alone) (Figure 3C). After co-culture with WJ-MSCs or BM-MSCs following OGD, both treated cultures showed a significant reduction in cell death and apoptosis compared to the OGD-injured alone group, with WJ-MSC group showed the lowest cell death and apoptosis rate (Figure 3C). The total neurite length and number of branch points at 72 hours post-OGD was increased significantly by co-culture with both BM-MSCs and WJ-MSCs compared to that of the OGD-injured alone group (Figure 3D). Moreover, the co-culture with WJ-MSCs produced the most increase in neurite outgrowth (Figure 3D). Neuronal proliferation was found to be increased in the WJ-MSC group but not in the BM-MSC group (Figure 3E, left panel). It is known that cerebral ischemia also induces astrocyte reactivation, and the formation of glial scar hampers brain recovery [44]. In our study, astrocytes proliferated to approximately 16% after OGD. The number of astrocytes was only reduced in the WJ-MSC group, but not in the BM-MSC group (Figure 3E, right panel). These results showed that WJ-MSC secreted factors have better neuroprotective effects than those from BM-MSCs.

### WJ-MSC conditioned medium induces better angiogenesis-related abilities in endothelial cells

**Figure 4:**
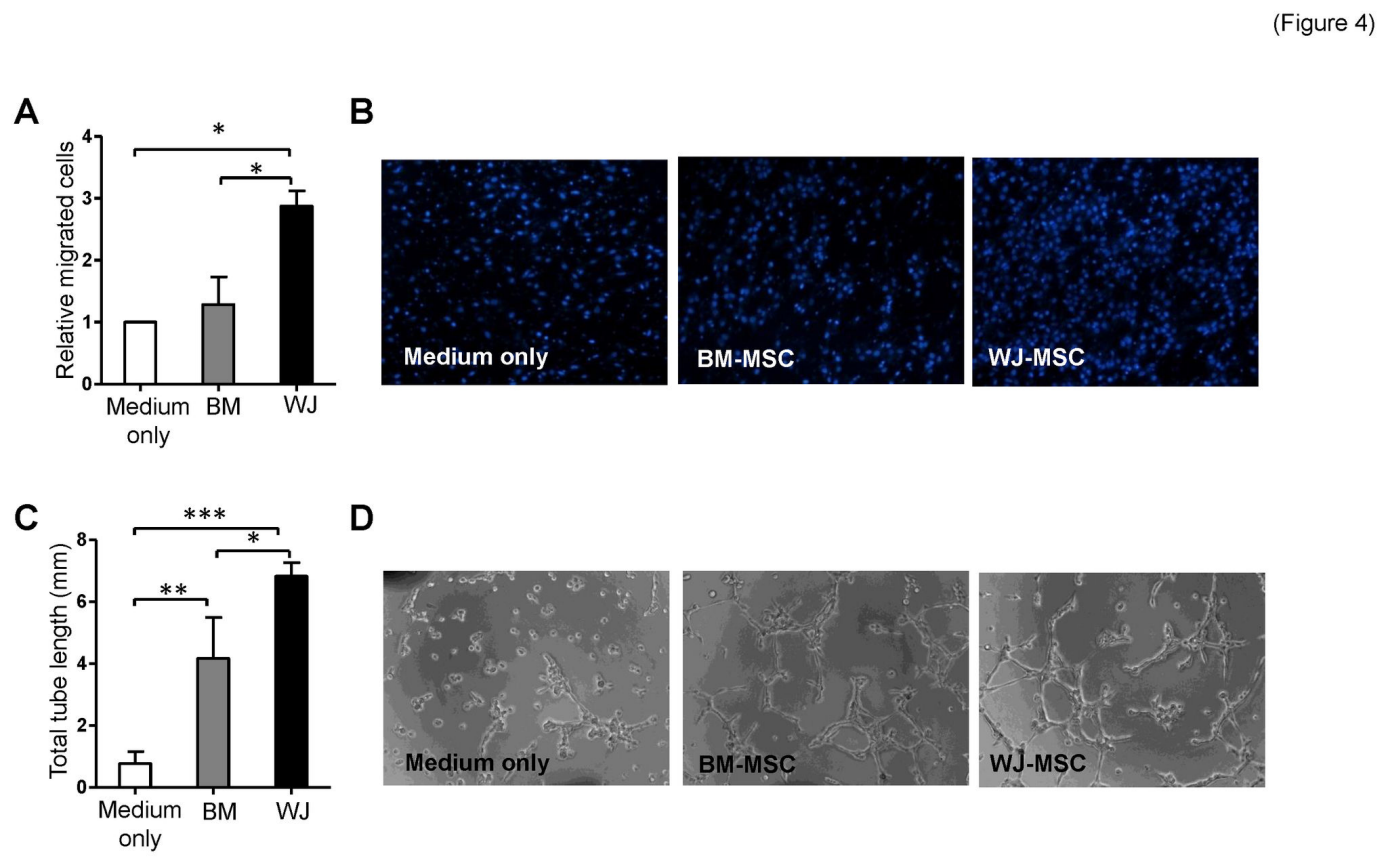
Preferential angiogenic induction ability of WJ-MSCs. (**A**–**B**) HMEC1 endothelial cells *in vitro* motility. Migrated HMEC1 that were attracted by BM-MSCs and WJ-MSCs were counted. MSC culture medium was used as negative control (medium only). Representative images of migrated HMEC1 cells are shown (B). (**C**–**D**) HMEC1 cells *in vitro* tube formation using Matrigel at 4 hours incubation in conditioned medium from BM-MSCs or conditioned medium from WJ-MSCs. Representative images of the HMEC1 tube formation are shown (D). (*p<0.05, **p<0.01, ***p<0.001).

To determine the angiogenic induction abilities of conditioned media of these two types of MSCs, we performed cell migration and tube formation assays on co-cultured HMEC1 human microvascular endothelial cells. As shown in Figure 4A-B, more HMEC1 cells moved toward WJ-MSCs than towards the BM-MSCs or medium only using Transwell assays. The ability of HMEC1 cells to form microvasculature *in vitro* were promoted significantly after cultured with MSC conditioned medium (Figure 4C-D) and, importantly, the total tube length generated by HMEC1 cells was longer when incubated with conditioned medium from WJ-MSCs (Figure 4C) compared to when incubated with conditioned medium from BM-MSCs.

## Discussion

It is clear that MSCs derived from various tissues exhibit distinct properties, which may influence their potential applications. Genome-wide approaches have unveiled the unique properties of MSCs efficiently [16,45,46]. By systems biology analysis, it was shown that WJ-MSCs and BM-MSCs possess distinct osteogenic and stemness potentials. Specifically, WJ-MSCs express more stemness and growth related genes, whereas BM-MSCs express more genes involved in skeletal development [36]. Translating preclinical testing to human trials is made more complex by various considerations, namely the delivery method, the type of stem cell selected, and the relationship between dose and cell survival. Although much progress has been made in stem cell biology, the clinical application of these cells is stymied by an inadequate or insufficient understanding of the functional integration of their complex interactive genetic processes. Further characterization of stem cells *via* a genomics approach is critical to obtaining a better understanding of the molecular parameters involved in cell-based therapy.

Although MSCs may repair damaged brain tissue or endothelium by direct differentiation, they also have a second function by secreting trophic factors that allow the recruitment of endogenous stem/precursor cells, which helps to facilitate disease recovery. Previous studies have shown that although transplanted BM-MSCs are able to improve cerebral ischemia *in vivo*, only a few of these MSCs differentiated into neurons (~2%) or astrocytes (~3%) [47]. Injected MSCs seem also to act *via* a paracrine mechanism and secrete trophic factors; this will inhibit scar formation (mainly caused by astrocytosis) as well as stimulating neural progenitor cells (NPCs) proliferation, migration and differentiation [48,49]. The growth and trophic factors secreted by the injected MSCs are able to enhance angiogenesis, synaptogenesis, and neurogenesis [50]. As to mechanisms, it has been shown that conditioned medium of human bone marrow MSCs (BM-MSCs) can activate the PI3K-Akt pathway in hypoxic endothelial cells resulting in an inhibition of apoptosis, an increased cell survival, and a stimulation of angiogenesis; this is believed to be partly due to these MSCs having a higher content of anti-apoptotic and angiogenic factors, such as IL-6, VEGF, and monocyte chemoattractant protein (MCP)-1 [51]. As yet, in this context, the paracrine factors of WJ-MSCs remain to be elucidated. MSCs from umbilical cord blood are also able to increase the expression levels of local neurotransmitters, such as brain-derived neurotrophic factor (BDNF), neurotrophin-3 (NTF3), which should enhance disease recovery [52]. When human umbilical mesenchymal stem cells were used to cure ischemic stroke in rats, implanted WJ-MSCs were shown to survive in the infarct cortex for at least 36 days and released neuroprotective and growth-associated cytokines including BDNF, platelet-derived growth factor-AA, angiopoietin-2, CXCL-16, and neutrophil-activating protein-2 [53]. It was also suggested that the transplanted neural progenitors derived from human WJ-MSCs was less likely to directly integrate into the neuronal network of the rat host, but rather released the aforementioned nourishing factors to enhance indirectly the endogenous tissue repair as did the undifferentiated WJ-MSCs [53]. A similar scenario also occurs during NPC transplantation: NPCs are able to accelerate CNS regeneration by secreting various neurotrophic cytokines such as BDNF and NTF3, and nerve growth factor (NGF) [54]. These secreted cytokines promote axon growth and increase the neuroplasticity of existing neurons, and therefore may be another therapeutic mechanism associated with NPC engraftment [54].

The neurogenesis and angiogenesis effects of BM-MSC and WJ-MSC secretomes have not been well compared at a side-by-side manner, although a similar vis-à-vis study has been performed on human MSCs derived from bone marrow, adipose (ASCs), and dermal tissue [28]. Here we provided a comprehensive list of the differentially secreted factors produced by the two types of MSCs examined in the present study, as well as the correlation of the factors with the neurogenesis and angiogenesis potentials of two MSC types (Figure 2A). Differential expression by the two types of MSCs should lead to differences in their ability to induce neurogenesis, neuroprotection, and angiogenesis (Figures 2-4). Specifically, NTF3, EGF, MDK, HBEGF, CXCL2, CXCL5 and FGF9 are more abundant in WJ-MSCs. Among these factors, NTF3, EGF, HBEGF and MDK are known to be involved in neurogenesis, while NTF3 is a neurotrophic factor that helps neuron survival and differentiation. EGF is able to enhance neuron differentiation and maturation [55], and midkine (MDK) is known to be involved in neuronal precursor cell growth [56]. Finally, HBEGF is induced after hypoxic or ischemic injury and has been shown to stimulate neurogenesis in the adult brain [57]. The abundant expression of trophic factors by WJ-MSCs compared to BM-MSCs is able to partly explained why N2a cells, when co-cultured with MSCs, migration towards WJ-MSCs more rapidly than towards BM-MSCs and why N2a cells differentiate into TUBB3-positive neurons more efficiently in the presence of conditioned medium from WJ-MSCs compared to conditioned medium from BM-MSCs (Figure 2). It also helps to explain why OGD-injured rat cortical neurons survive better when co-cultured with WJ-MSCs than with BM-MSCs (Figure 3).

MDK is also an angiogenic factor that enhances endothelial proliferation and increases tumor vascular density [58]. EGF and HBEGF belong to the EGF superfamily. EGF is present in normal human endothelium and is important for blood vessel functionality [59]. In addition, both HBEGF and EGF stimulate human umbilical vein endothelial cells (HUVEC) migration and promote tube formation *via* activation of the PI3K and MAPK signaling pathways [60]. CXCL2 and CXCL5, which are potent promoters of angiogenesis, are members of the CXC chemokine family and have the Glutamic acid–Leucine–Arginine (ELR) motif. Both CXCL2 and CXCL5 regulate angiogenesis *via* binding to and activating CXCR2 present in endothelium [61]. These differentially expressed angiogenic factors may contribute to why WJ-MSCs are able to induce endothelial cells migrate more efficiently than BM-MSCs do and WJ-MSC conditioned medium is also able to promote greater microvasculature formation than BM-MSC conditioned medium. Whether WJ-MSCs are also able to confer better therapeutic effects *in vivo* is currently under investigation.

Our results confirmed that WJ-MSCs secreted highly levels of CXCL5 compared with BM-MSCs. Administration of CXCL5 can enhance HUVEC proliferation, invasion and tube formation abilities [62]. CXCL5 has been reported as an angiogenic factor in non-small cell lung cancer, where the protein level of CXCL5 is positively correlated with tumor vessel density [63]. CXCL5 secreted by rat adipose tissue-derived stem cells also has neurotrophic effects on rat major pelvic ganglia [64]. However, secreted levels of CXCL5 were hard to be detected in human BM-MSC and adipose tissue MSC conditioned medium [28]. The importance of CXCL5 in WJ-MSC conditioned medium related to neuroprotective, neurogenesis and angiogenesis will need further investigation.

An improved understanding of MSCs from different sources will help the clinical application of these cells and allow control of their differentiation *in vivo*. This will help their future potential when applied clinically. Studying the paracrine factors that are differentially produced in various types of MSCs should eventually allow us to design new therapeutic approaches based on different MSCs. Thus, our findings contribute new insights that refine our molecular picture of MSC subtypes and help to provide a better understanding of the two MSC subtypes investigated in the present study.

## Supporting Information

Table S1 Primers used in real-time PCR experiments. (PDF)

Table S2 Genes that differentially expressed in BM-MSCs and WJ-MSCs. (XLSX)

## References

[1] Horwitz EM, Prockop DJ, Fitzpatrick LA, Koo WW, Gordon PL et al. (1999) Transplantability and therapeutic effects of bone marrow-derived mesenchymal cells in children with osteogenesis imperfecta. Nat Med 5: 309-313. doi:10.1038/6529. PubMed: 10086387. 10086387 10.1038/6529

[2] Kawada H, Fujita J, Kinjo K, Matsuzaki Y, Tsuma M et al. (2004) Nonhematopoietic mesenchymal stem cells can be mobilized and differentiate into cardiomyocytes after myocardial infarction. Blood 104: 3581-3587. doi:10.1182/blood-2004-04-1488. PubMed: 15297308. 15297308 10.1182/blood-2004-04-1488

[3] Ripa RS, Haack-Sørensen M, Wang Y, Jørgensen E, Mortensen S et al. (2007) Bone marrow derived mesenchymal cell mobilization by granulocyte-colony stimulating factor after acute myocardial infarction: results from the Stem Cells in Myocardial Infarction (STEMMI) trial. Circulation 116: I24-I30. PubMed: 17846310. 17846310 10.1161/CIRCULATIONAHA.106.678649

[4] Bang OY, Lee JS, Lee PH, Lee G (2005) Autologous mesenchymal stem cell transplantation in stroke patients. Ann Neurol 57: 874-882. doi:10.1002/ana.20501. PubMed: 15929052. 15929052 10.1002/ana.20501

[5] Chen J, Li Y, Wang L, Zhang Z, Lu D et al. (2001) Therapeutic benefit of intravenous administration of bone marrow stromal cells after cerebral ischemia in rats. Stroke 32: 1005-1011. doi:10.1161/01.STR.32.4.1005. PubMed: 11283404. 11283404 10.1161/01.str.32.4.1005

[6] Lee JS, Hong JM, Moon GJ, Lee PH, Ahn YH et al. (2010) A long-term follow-up study of intravenous autologous mesenchymal stem cell transplantation in patients with ischemic stroke. Stem Cells 28: 1099-1106. doi:10.1002/stem.430. PubMed: 20506226. 20506226 10.1002/stem.430

[7] Ra JC, Shin IS, Kim SH, Kang SK, Kang BC et al. (2011) Safety of intravenous infusion of human adipose tissue-derived mesenchymal stem cells in animals and humans. Stem Cells Dev 20: 1297-1308. doi:10.1089/scd.2010.0466. PubMed: 21303266. 21303266 10.1089/scd.2010.0466

[8] Xiong Y, Mahmood A, Chopp M (2010) Angiogenesis, neurogenesis and brain recovery of function following injury. Curr Opin Investig Drugs 11: 298-308. PubMed: 20178043. PMC2836170 20178043

[9] Lee OK, Kuo TK, Chen WM, Lee KD, Hsieh SL et al. (2004) Isolation of multipotent mesenchymal stem cells from umbilical cord blood. Blood 103: 1669-1675. doi:10.1182/blood-2003-05-1670. PubMed: 14576065. 14576065 10.1182/blood-2003-05-1670

[10] Panepucci RA, Siufi JL, Silva WA Jr., Proto-Siquiera R, Neder L et al. (2004) Comparison of gene expression of umbilical cord vein and bone marrow-derived mesenchymal stem cells. Stem Cells 22: 1263-1278. doi:10.1634/stemcells.2004-0024. PubMed: 15579645. 15579645 10.1634/stemcells.2004-0024

[11] Chang YJ, Shih DT, Tseng CP, Hsieh TB, Lee DC et al. (2006) Disparate mesenchyme-lineage tendencies in mesenchymal stem cells from human bone marrow and umbilical cord blood. Stem Cells 24: 679-685. doi:10.1634/stemcells.2004-0308. PubMed: 16179428. 16179428 10.1634/stemcells.2004-0308

[12] Prockop DJ (1997) Marrow stromal cells as stem cells for nonhematopoietic tissues. Science 276: 71-74. doi:10.1126/science.276.5309.71. PubMed: 9082988. 9082988 10.1126/science.276.5309.71

[13] Wang HS, Hung SC, Peng ST, Huang CC, Wei HM et al. (2004) Mesenchymal stem cells in the Wharton’s jelly of the human umbilical cord. Stem Cells 22: 1330-1337. doi:10.1634/stemcells.2004-0013. PubMed: 15579650. 15579650 10.1634/stemcells.2004-0013

[14] Mitchell KE, Weiss ML, Mitchell BM, Martin P, Davis D et al. (2003) Matrix cells from Wharton’s jelly form neurons and glia. Stem Cells 21: 50-60. doi:10.1634/stemcells.21-1-50. PubMed: 12529551. 12529551 10.1634/stemcells.21-1-50

[15] Balasubramanian S, Venugopal P, Sundarraj S, Zakaria Z, Majumdar AS et al. (2012) Comparison of chemokine and receptor gene expression between Wharton’s jelly and bone marrow-derived mesenchymal stromal cells. Cytotherapy 14: 26-33. doi:10.3109/14653249.2011.605119. PubMed: 22091833. 22091833 10.3109/14653249.2011.605119

[16] Al-Nbaheen M, Vishnubalaji R, Ali D, Bouslimi A, Al-Jassir F et al. (2012) Human Stromal (Mesenchymal) Stem Cells from Bone Marrow, Adipose Tissue and Skin Exhibit Differences in Molecular Phenotype and Differentiation Potential. Stem. Cell Res. 10.1007/s12015-012-9365-8 PMC3563956 22529014

[17] Kern S, Eichler H, Stoeve J, Kluter H, Bieback K (2006) Comparative Analysis of Mesenchymal Stem Cells from Bone Marrow, Umbilical Cord Blood or Adipose Tissue. Stem Cells. 10.1634/stemcells.2005-0342 16410387

[18] Strioga M, Viswanathan S, Darinskas A, Slaby O, Michalek J (2012) Same or not the same? Comparison of adipose tissue-derived versus bone marrow-derived mesenchymal stem and stromal cells. Stem Cells Dev 21: 2724-2752. doi:10.1089/scd.2011.0722. PubMed: 22468918. 22468918 10.1089/scd.2011.0722

[19] Miranda HC, Herai RH, Thomé CH, Gomes GG, Panepucci RA et al. (2012) A quantitative proteomic and transcriptomic comparison of human mesenchymal stem cells from bone marrow and umbilical cord vein. Proteomics 12: 2607-2617. doi:10.1002/pmic.201200111. PubMed: 22778083. 22778083 10.1002/pmic.201200111

[20] Liao W, Xie J, Zhong J, Liu Y, Du L et al. (2009) Therapeutic effect of human umbilical cord multipotent mesenchymal stromal cells in a rat model of stroke. Transplantation 87: 350-359. doi:10.1097/TP.0b013e318195742e. PubMed: 19202439. 19202439 10.1097/TP.0b013e318195742e

[21] Zhao LR, Duan WM, Reyes M, Keene CD, Verfaillie CM et al. (2002) Human bone marrow stem cells exhibit neural phenotypes and ameliorate neurological deficits after grafting into the ischemic brain of rats. Exp Neurol 174: 11-20. doi:10.1006/exnr.2001.7853. PubMed: 11869029. 11869029 10.1006/exnr.2001.7853

[22] Chen J, Chopp M (2006) Neurorestorative treatment of stroke: cell and pharmacological approaches. NeuroRx 3: 466-473. doi:10.1016/j.nurx.2006.07.007. PubMed: 17012060. 17012060 10.1016/j.nurx.2006.07.007 PMC2790719

[23] Ranganath SH, Levy O, Inamdar MS, Karp JM (2012) Harnessing the mesenchymal stem cell secretome for the treatment of cardiovascular disease. Cell Stem Cell 10: 244-258. doi:10.1016/j.stem.2012.02.005. PubMed: 22385653. 22385653 10.1016/j.stem.2012.02.005 PMC3294273

[24] Carvalho MM, Teixeira FG, Reis RL, Sousa N, Salgado AJ (2011) Mesenchymal stem cells in the umbilical cord: phenotypic characterization, secretome and applications in central nervous system regenerative medicine. Curr Stem Cell Res Ther 6: 221-228. doi:10.2174/157488811796575332. PubMed: 21476975. 21476975 10.2174/157488811796575332

[25] Ribeiro CA, Salgado AJ, Fraga JS, Silva NA, Reis RL et al. (2011) The secretome of bone marrow mesenchymal stem cells-conditioned media varies with time and drives a distinct effect on mature neurons and glial cells (primary cultures). J Tissue Eng Regen Med 5: 668-672. doi:10.1002/term.365. PubMed: 21774090. 21774090 10.1002/term.365

[26] Tsai CC, Chen YJ, Yew TL, Chen LL, Wang JY et al. (2011) Hypoxia inhibits senescence and maintains mesenchymal stem cell properties through down-regulation of E2A-p21 by HIF-TWIST. Blood 117: 459-469. doi:10.1182/blood-2010-05-287508. PubMed: 20952688. 20952688 10.1182/blood-2010-05-287508

[27] Oskowitz A, McFerrin H, Gutschow M, Carter ML, Pochampally R (2011) Serum-deprived human multipotent mesenchymal stromal cells (MSCs) are highly angiogenic. Stem Cell Res 6: 215-225. doi:10.1016/j.scr.2011.01.004. PubMed: 21421339. 21421339 10.1016/j.scr.2011.01.004 PMC4920087

[28] Hsiao ST, Asgari A, Lokmic Z, Sinclair R, Dusting GJ et al. (2012) Comparative analysis of paracrine factor expression in human adult mesenchymal stem cells derived from bone marrow, adipose, and dermal tissue. Stem Cells Dev 21: 2189-2203. doi:10.1089/scd.2011.0674. PubMed: 22188562. 22188562 10.1089/scd.2011.0674 PMC3411362

[29] Ribeiro CA, Fraga JS, Grãos M, Neves NM, Reis RL et al. (2012) The secretome of stem cells isolated from the adipose tissue and Wharton jelly acts differently on central nervous system derived cell populations. Stem Cell Res Ther 3: 18. doi:10.1186/scrt109. PubMed: 22551705. 22551705 10.1186/scrt109 PMC3392765

[30] Fu YS, Cheng YC, Lin MY, Cheng H, Chu PM et al. (2006) Conversion of human umbilical cord mesenchymal stem cells in Wharton’s jelly to dopaminergic neurons in vitro: potential therapeutic application for Parkinsonism. Stem Cells 24: 115-124. doi:10.1634/stemcells.2005-0053. PubMed: 16099997. 16099997 10.1634/stemcells.2005-0053

[31] Wang HW, Trotter MW, Lagos D, Bourboulia D, Henderson S et al. (2004) Kaposi sarcoma herpesvirus-induced cellular reprogramming contributes to the lymphatic endothelial gene expression in Kaposi sarcoma. Nat Genet 36: 687-693. doi:10.1038/ng1384. PubMed: 15220918. 15220918 10.1038/ng1384

[32] Huang TS, Hsieh JY, Wu YH, Jen CH, Tsuang YH et al. (2008) Functional network reconstruction reveals somatic stemness genetic maps and dedifferentiation-like transcriptome reprogramming induced by GATA2. Stem Cells 26: 1186-1201. doi:10.1634/stemcells.2007-0821. PubMed: 18308945. 18308945 10.1634/stemcells.2007-0821

[33] Yang TP, Chang TY, Lin CH, Hsu MT, Wang HW (2006) ArrayFusion: a web application for multi-dimensional analysis of CGH, SNP and microarray data. Bioinformatics 22: 2697-2698. doi:10.1093/bioinformatics/btl457. PubMed: 16935928. 16935928 10.1093/bioinformatics/btl457

[34] Dennis G Jr., Sherman BT, Hosack DA, Yang J, Gao W et al. (2003) DAVID: Database for Annotation, Visualization, and Integrated Discovery. Genome Biol 4: 3. doi:10.1186/gb-2003-4-5-p3. PMC193660 12734009

[35] Mattson MP, Barger SW, Begley JG, Mark RJ (1995) Calcium, free radicals, and excitotoxic neuronal death in primary cell culture. Methods Cell Biol 46: 187-216. doi:10.1016/S0091-679X(08)61930-5. PubMed: 7541884. 7541884 10.1016/s0091-679x(08)61930-5

[36] Hsieh JY, Fu YS, Chang SJ, Tsuang YH, Wang HW (2010) Functional module analysis reveals differential osteogenic and stemness potentials in human mesenchymal stem cells from bone marrow and Wharton’s jelly of umbilical cord. Stem Cells Dev 19: 1895-1910. doi:10.1089/scd.2009.0485. PubMed: 20367285. 20367285 10.1089/scd.2009.0485

[37] Harris MA, Clark J, Ireland A, Lomax J, Ashburner M et al. (2004) The Gene Ontology (GO) database and informatics resource. Nucleic Acids Res 32: D258-D261. doi:10.1093/nar/gkh036. PubMed: 14681407. 14681407 10.1093/nar/gkh036 PMC308770

[38] Jankowski MP, Cornuet PK, McIlwrath S, Koerber HR, Albers KM (2006) SRY-box containing gene 11 (Sox11) transcription factor is required for neuron survival and neurite growth. Neuroscience 143: 501-514. doi:10.1016/j.neuroscience.2006.09.010. PubMed: 17055661. 17055661 10.1016/j.neuroscience.2006.09.010 PMC1698553

[39] Freed WJ, Chen J, Bäckman CM, Schwartz CM, Vazin T et al. (2008) Gene expression profile of neuronal progenitor cells derived from hESCs: activation of chromosome 11p15.5 and comparison to human dopaminergic neurons. PLOS ONE 3: e1422. doi:10.1371/journal.pone.0001422. PubMed: 18183302. 18183302 10.1371/journal.pone.0001422 PMC2170519

[40] Mahlapuu M, Ormestad M, Enerbäck S, Carlsson P (2001) The forkhead transcription factor Foxf1 is required for differentiation of extra-embryonic and lateral plate mesoderm. Development 128: 155-166. PubMed: 11124112. 11124112 10.1242/dev.128.2.155

[41] Astorga J, Carlsson P (2007) Hedgehog induction of murine vasculogenesis is mediated by Foxf1 and Bmp4. Development 134: 3753-3761. doi:10.1242/dev.004432. PubMed: 17881493. 17881493 10.1242/dev.004432

[42] Wu PY, Lin YC, Chang CL, Lu HT, Chin CH et al. (2009) Functional decreases in P2X7 receptors are associated with retinoic acid-induced neuronal differentiation of Neuro-2a neuroblastoma cells. Cell Signal 21: 881-891. doi:10.1016/j.cellsig.2009.01.036. PubMed: 19385050. 19385050 10.1016/j.cellsig.2009.01.036

[43] Zeng M, Zhou JN (2008) Roles of autophagy and mTOR signaling in neuronal differentiation of mouse neuroblastoma cells. Cell Signal 20: 659-665. doi:10.1016/j.cellsig.2007.11.015. PubMed: 18207367. 18207367 10.1016/j.cellsig.2007.11.015

[44] Pekny M, Nilsson M (2005) Astrocyte activation and reactive gliosis. Glia 50: 427-434. doi:10.1002/glia.20207. PubMed: 15846805. 15846805 10.1002/glia.20207

[45] Chen HC, Lee YS, Sieber M, Lu HT, Wei PC et al. (2012) MicroRNA and messenger RNA analyses of mesenchymal stem cells derived from teeth and the Wharton jelly of umbilical cord. Stem Cells Dev 21: 911-922. doi:10.1089/scd.2011.0186. PubMed: 21732813. 21732813 10.1089/scd.2011.0186

[46] Tsai MS, Hwang SM, Chen KD, Lee YS, Hsu LW et al. (2007) Functional network analysis of the transcriptomes of mesenchymal stem cells derived from amniotic fluid, amniotic membrane, cord blood, and bone marrow. Stem Cells 25: 2511-2523. doi:10.1634/stemcells.2007-0023. PubMed: 17556597. 17556597 10.1634/stemcells.2007-0023

[47] Chen J, Li Y, Wang L, Lu M, Zhang X et al. (2001) Therapeutic benefit of intracerebral transplantation of bone marrow stromal cells after cerebral ischemia in rats. J Neurol Sci 189: 49-57. doi:10.1016/S0022-510X(01)00557-3. PubMed: 11535233. 11535233 10.1016/s0022-510x(01)00557-3

[48] Chen J, Li Y, Katakowski M, Chen X, Wang L et al. (2003) Intravenous bone marrow stromal cell therapy reduces apoptosis and promotes endogenous cell proliferation after stroke in female rat. J Neurosci Res 73: 778-786. doi:10.1002/jnr.10691. PubMed: 12949903. 12949903 10.1002/jnr.10691

[49] Bai L, Caplan A, Lennon D, Miller RH (2007) Human mesenchymal stem cells signals regulate neural stem cell fate. Neurochem Res 32: 353-362. doi:10.1007/s11064-006-9212-x. PubMed: 17191131. 17191131 10.1007/s11064-006-9212-x

[50] Chopp M, Li Y (2002) Treatment of neural injury with marrow stromal cells. Lancet Neurol 1: 92-100. doi:10.1016/S1474-4422(02)00040-6. PubMed: 12849513. 12849513 10.1016/s1474-4422(02)00040-6

[51] Hung SC, Pochampally RR, Chen SC, Hsu SC, Prockop DJ (2007) Angiogenic effects of human multipotent stromal cell conditioned medium activate the PI3K-Akt pathway in hypoxic endothelial cells to inhibit apoptosis, increase survival, and stimulate angiogenesis. Stem Cells 25: 2363-2370. doi:10.1634/stemcells.2006-0686. PubMed: 17540857. 17540857 10.1634/stemcells.2006-0686

[52] Dasari VR, Spomar DG, Gondi CS, Sloffer CA, Saving KL et al. (2007) Axonal remyelination by cord blood stem cells after spinal cord injury. J Neurotrauma 24: 391-410. doi:10.1089/neu.2006.0142. PubMed: 17376002. 17376002 10.1089/neu.2006.0142 PMC1859845

[53] Lin YC, Ko TL, Shih YH, Lin MY, Fu TW et al. (2011) Human umbilical mesenchymal stem cells promote recovery after ischemic stroke. Stroke 42: 2045-2053. doi:10.1161/STROKEAHA.110.603621. PubMed: 21566227. 21566227 10.1161/STROKEAHA.110.603621

[54] Kamei N, Tanaka N, Oishi Y, Hamasaki T, Nakanishi K et al. (2007) BDNF, NT-3, and NGF released from transplanted neural progenitor cells promote corticospinal axon growth in organotypic cocultures. Spine 32: 1272-1278. doi:10.1097/BRS.0b013e318059afab. PubMed: 17515814. 17515814 10.1097/BRS.0b013e318059afab

[55] Wong RW, Guillaud L (2004) The role of epidermal growth factor and its receptors in mammalian CNS. Cytokine Growth Factor Rev 15: 147-156. doi:10.1016/j.cytogfr.2004.01.004. PubMed: 15110798. 15110798 10.1016/j.cytogfr.2004.01.004

[56] Zou P, Muramatsu H, Miyata T, Muramatsu T (2006) Midkine, a heparin-binding growth factor, is expressed in neural precursor cells and promotes their growth. J Neurochem 99: 1470-1479. doi:10.1111/j.1471-4159.2006.04138.x. PubMed: 17230638. 17230638 10.1111/j.1471-4159.2006.04138.x

[57] Jin K, Mao XO, Sun Y, Xie L, Jin L et al. (2002) Heparin-binding epidermal growth factor-like growth factor: hypoxia-inducible expression in vitro and stimulation of neurogenesis in vitro and in vivo. J Neurosci 22: 5365-5373. PubMed: 12097488. 12097488 10.1523/JNEUROSCI.22-13-05365.2002 PMC6758221

[58] Choudhuri R, Zhang HT, Donnini S, Ziche M, Bicknell R (1997) An angiogenic role for the neurokines midkine and pleiotrophin in tumorigenesis. Cancer Res 57: 1814-1819. PubMed: 9135027. 9135027

[59] Möller B, Rasmussen C, Lindblom B, Olovsson M (2001) Expression of the angiogenic growth factors VEGF, FGF-2, EGF and their receptors in normal human endometrium during the menstrual cycle. Mol Hum Reprod 7: 65-72. doi:10.1093/molehr/7.1.65. PubMed: 11134362. 11134362 10.1093/molehr/7.1.65

[60] Mehta VB, Besner GE (2007) HB-EGF promotes angiogenesis in endothelial cells via PI3-kinase and MAPK signaling pathways. Growth Factors 25: 253-263. doi:10.1080/08977190701773070. PubMed: 18092233. 18092233 10.1080/08977190701773070

[61] Strieter RM, Belperio JA, Burdick MD, Keane MP (2005) CXC chemokines in angiogenesis relevant to chronic fibroproliferation. Curr Drug Targets Inflamm Allergy 4: 23-26. doi:10.2174/1568010053622902. PubMed: 15720231. 15720231 10.2174/1568010053622902

[62] Matsuo Y, Raimondo M, Woodward TA, Wallace MB, Gill KR et al. (2009) CXC-chemokine/CXCR2 biological axis promotes angiogenesis in vitro and in vivo in pancreatic cancer. Int J Cancer 125: 1027-1037. doi:10.1002/ijc.24383. PubMed: 19431209. 19431209 10.1002/ijc.24383

[63] Arenberg DA, Keane MP, DiGiovine B, Kunkel SL, Morris SB et al. (1998) Epithelial-neutrophil activating peptide (ENA-78) is an important angiogenic factor in non-small cell lung cancer. J Clin Invest 102: 465-472. doi:10.1172/JCI3145. PubMed: 9691082. 9691082 10.1172/JCI3145 PMC508906

[64] Zhang H, Yang R, Wang Z, Lin G, Lue TF et al. (2011) Adipose tissue-derived stem cells secrete CXCL5 cytokine with neurotrophic effects on cavernous nerve regeneration. J Sex Med 8: 437-446. doi:10.1111/j.1743-6109.2010.02128.x. PubMed: 21114767. 21114767 10.1111/j.1743-6109.2010.02128.x PMC3176296

