# Supplementary material for: Mesenchymal Stem Cells from Human Umbilical Cord Express Preferentially Secreted Factors Related to Neuroprotection, Neurogenesis, and Angiogenesis
Source: PLoS One. 2013 Aug 22;8(8):e72604. doi: 10.1371/journal.pone.0072604 (PMC3749979; doi:10.1371/journal.pone.0072604)
Supplement: Table S1 — (PDF) [file pone.0072604.s001.pdf]

Table S1. Primers used in real-time PCR experiments.

Genes	Forward primer	Reverse primer	Amplicon
**SOX11**	CgCgggCAAgACggTCAAgT	CCTCCTCgTCCggCTCCTgT	104
**PITX1**	CgAgTCgTCTgACACggAg	TTCTTCTTggCTgggTCgTC	117
**NRP2**	ggCTggACCCCCAACTTg	TggAAATCgCTCCCTgTgTT	100
**FOXF1**	CAgCCgTATCTgCACCAgAA	ACTCCTTTCggTCACACATgCT	100
**FLT1**	CCATggTCAgCTACTgggAC	CTTgCATgATgTgCTgggTg	135
**CDH2**	CCACgCTgAgCCCCAgTATC	CCCCCAgTCgTCAggTAATCA	232
**PGF**	AATgTCACCATgCAgCTCCT	ACCTTTCCggCTTCATCTTCT	124
**ANGPT1**	gggAACCgAgCCTATTCACA	gCTgCTCTgTTTTCCTgCTg	108
**CXCL2**	ACCgAAgTCATAgCCACACTCA	TCAggAACAgCCACCAATAAgC	150
**HBEGF**	AgTCCgTgACTTgCAAgAgg	TTCTTTTCCCgTgCTCCTCC	104
**CXCL5**	CAgACCACgCAAggAgTTCATC	TCTTCAgggAggCTACCACTTC	100
**NTF3**	CggATgCCATggTTACTTTTg	CCTTggATgCCACggAgATA	100
**MDK**	TACAATgCTCAgTgCCAggA	CTTggCgTCTAgTCCTTTCC	107
**EGF**	CTCCTCCAAgTgCATCAACA	gCCTCCCTCTgTATTTgTgC	154
**FGF9**	ACACTggAAggCgATACTATgTTgC	TACTTTgTCggggTCCACTggTCT	122
**GAPDH**	ggAgTCCACTggCgTCTTCA	TggTTCACACCCATgACgAA	123
